# The potential interplay between G-quadruplex and p53: their roles in regulation of ferroptosis in cancer

**DOI:** 10.3389/fmolb.2022.965924

**Published:** 2022-07-25

**Authors:** Lulu Zhang, Yi Lu, Xiaoli Ma, Yuanxin Xing, Jinbo Sun, Yanfei Jia

**Affiliations:** ^1^ Research Center of Basic Medicine, Jinan Central Hospital, Shandong First Medical University, Jinan, China; ^2^ Research Center of Basic Medicine, Jinan Central Hospital, Shandong University, Jinan, China; ^3^ Department of Neurology, Jinan Central Hospital, Shandong University, Jinan, China

**Keywords:** G-quadruplex, p53, ferroptosis, cancer, biological activities

## Abstract

Ferroptosis is a novel form of regulated cell death trigged by various biological processes, and p53 is involved in different ferroptosis regulations and functions as a crucial regulator. Both DNA and RNA can fold into G-quadruplex in GC-rich regions and increasing shreds of evidence demonstrate that G-quadruplexes have been associated with some important cellular events. Investigation of G-quadruplexes is thus vital to revealing their biological functions. Specific G-quadruplexes are investigated to discover new effective anticancer drugs. Multiple modulations have been discovered between the secondary structure G-quadruplex and p53, probably further influencing the ferroptosis in cancer. G-quadruplex binds to ferric iron-related structures directly and may affect the p53 pathways as well as ferroptosis in cancer. In addition, G-quadruplex also interacts with p53 indirectly, including iron-sulfur cluster metabolism, telomere homeostasis, lipid peroxidation, and glycolysis. In this review, we summarized the latent interplay between G-quadruplex and p53 which focused mainly on ferroptosis in cancer to provide the potential understanding and encourage future studies.

## Introduction

Ferroptosis is a newly identified type of regulated cell death that has attracted considerable attention in explaining the signaling pathways and defined effector mechanisms. Triggering cell death is one of the principal approaches to killing cancer cells. Emerging evidence shows cancer cells exhibit an increased iron demand compared with normal, non-cancer cells. This iron dependency can make cancer cells more vulnerable to ferroptosis. Therefore, exploiting how the ferroptosis are modulated could open new therapeutic avenues for eliminating cancer cells.

Although the precise molecular mechanism of ferroptosis has not been fully understood, many different genes involved in iron metabolism and lipid peroxidation, such as GSH peroxidase 4 (GPX4) and solute carrier family 7 member 11 (SLC7A11), have been shown to be the key regulators in ferroptosis (Chen X. et al., 2021). Recently, studies have demonstrated that p53 regulates ferroptosis through transcription-dependent and -independent mechanisms (Kang et al., 2019; Liu et al., 2019; Liu J. et al., 2020; Liu and Gu, 2022). P53 was discovered to bind to the promoter region of SLC7A11 to repress its expression (Jiang et al., 2015; Ou et al., 2016). The binding of p53 to dipeptidyl-peptidase-4 (DPP4) protein decreased lipid peroxidation and ferroptosis (Xie et al., 2017). PTGS2 expression was demonstrated to be upregulated by ferroptosis in a p53-dependent manner (Jiang et al., 2015). Conversely, p53 acts as a positive regulator of ferroptosis via regulation of cytochrome c oxidase 2 (SCO2), glucose transporter (GLUT)1, GLUT4 and glutaminase 2 (GLS2) (Schwartzenberg-Bar-Yoseph et al., 2004; Zhang et al., 2018). Besides, p53 suppresses the expression of RNA-binding protein ELAV-like RNA-binding protein 1 (ELAVL1), leading to impaired ELAVL1-LINC00336 interaction and further promoting ferroptosis (Wang et al., 2019).

**FIGURE 1:**
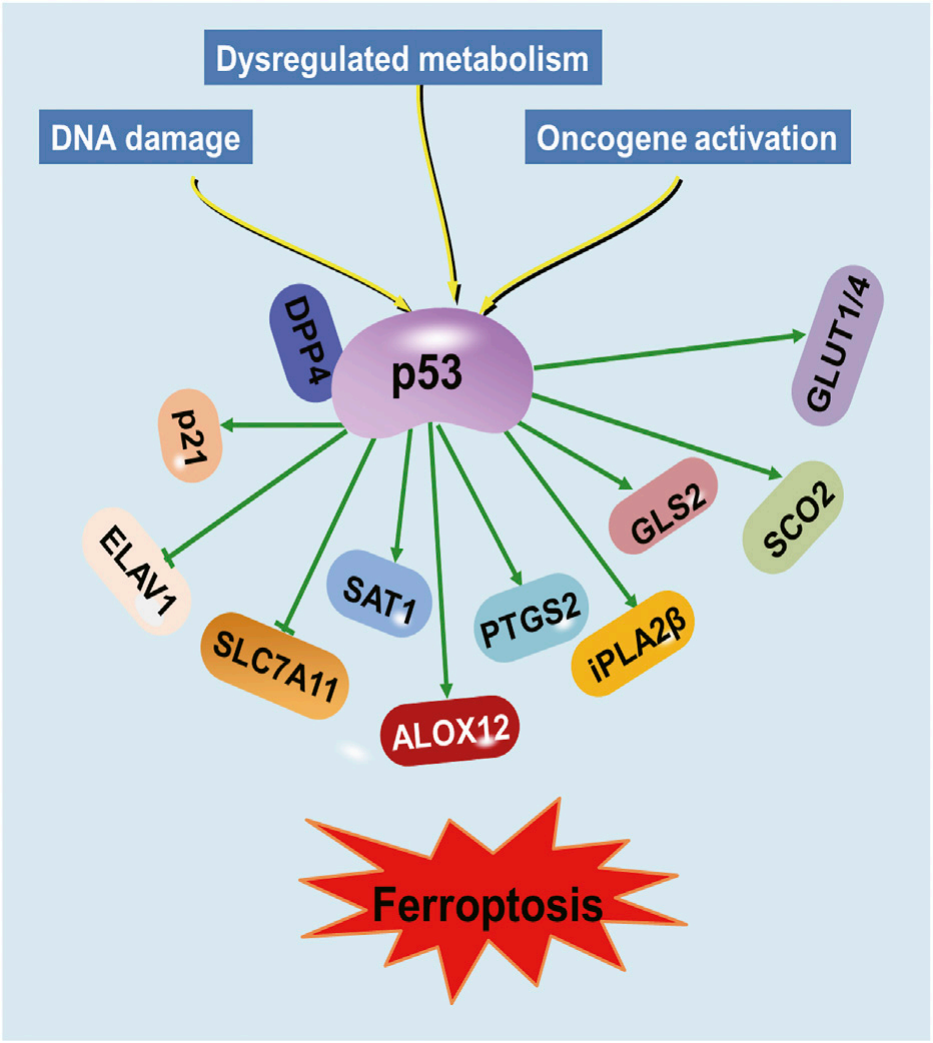
Regulation of ferroptosis by p53. Various proteins involved in iron metabolism and lipid peroxidation are modulated by p53.

Furthermore, p53’s role in the regulation of genes involved in metabolism has been implicated in its ability to regulate ferroptosis (Tarangelo et al., 2018; Liu and Gu, 2021; Yu et al., 2021). Arachidonate 12-lipooxygenase (ALOX12), a member of the lipoxygenase family that oxygenates polyunsaturated fatty acids (PUFAs), was identified as an important positive regulator for p53-mediated ferroptosis (Chu et al., 2019). The p53 suppresses ferroptosis through the induction of cyclin-dependent kinase inhibitor 1A (CDKN1A/p21) expression by suppressing the metabolic stress-induced ferroptosis (Tarangelo et al., 2018). The iPLA2β controls p53-driven ferroptosis by mediation detoxification of peroxidized lipids (Chen D. et al., 2021). Spermidine/spermine N1-acetyltransferase (SAT1) was also a direct p53 target that induced lipid peroxidation and sensitizes cells to undergo ferroptosis (Ou et al., 2016). Therefore, p53 represents a novel regulator of ferroptosis, an iron-catalyzed form of regulated necrosis that occurs through excessive peroxidation of PUFAs (Dixon et al., 2012) (Figure 1). An improved understanding of the molecular mechanisms and cellular factors of p53 in ferroptosis regulation will yield new therapeutic strategies for cancer.

Four guanines bind together through eight Hoogsteen hydrogen bonds to form G-quartet, and two or more G-quartets stack to become G-quadruplex (Yuan et al., 2011). Both DNA and RNA can fold into G-quadruplex in GC-rich regions, such as protomer regions, telomere regions, or UTR regions, and increasing shreds of evidence demonstrate the involvement of G-quadruplexes in different biological pathways (Spiegel et al., 2020; Varshney et al., 2020). The correction between G-quadruplex and cancer can be mainly described in the following three aspects. First, the promoter regions contain numerous G-rich sequences which can form G-quadruplexes (Shen et al., 2021). The G-quadruplex on the coding strand blocks the transcription complex and inhibits transcription, while the G-quadruplex on the non-coding strand helps to unwind the duplex and facilitate the transcription (Hänsel-Hertsch et al., 2016), hence the G-quadruplex functions in oncogene promoters have been well studied as well as ligands investigation (Siddiqui-Jain et al., 2002; Nasiri et al., 2014; Zhang et al., 2017). Second, as nucleoprotein complexes at the ends of chromosomes, the telomere is essential for chromosome stability and genome integrity. The repetitive G-rich sequences in telomere fold into G-quadruplex and block the telomeric elongation in cancer cells, thus the G-quadruplex is an effective target for tumor suppression and different G-quadruplex ligands are being developed to inhibit telomerase activity in cancer (Tauchi et al., 2003; Zhu et al., 2012; Beniaminov et al., 2016). Third, the genome instability is regulated by G-quadruplex through DNA replication (Rhodes and Lipps, 2015), leading to apoptosis and autophagy of cancer cells, and the ligand study in this area has been well-developed (Bywater et al., 2012; Xu et al., 2017; Beauvarlet et al., 2019). All these regulations support G-quadruplex as the anti-cancer target using G-quadruplex binding ligands as a tool. Although the primary sequences of G-quadruplexes are all G-riched with similar lengths, the secondary structures in three-dimensional reveal diverse topologies, providing possibilities for targeting.

The p53 has been found to bind telomeric G-quadruplex directly through DNA binding domains (Adámik et al., 2016). Both full-length and C-terminal regions of p53 display strong binding with myc G-quadruplex in the NHEIII_1_ region (Petr et al., 2016). The binding between p53 and G-quadruplex participates in gene regulation, in addition, the G-quadruplex can in return modulate the p53 function. The p53 RNA G-quadruplex structure close to the poly(A) site recruits DHX36, RNA helicase maintaining pre-mRNA 3′-end processing after UV damage (Newman et al., 2017), and the G-quadruplex structures formed in the GC-riched region of p53 intron 3 can regulate the splicing of p53 intron 2 (Marcel et al., 2010). Moreover, as part of the non-B DNA structure suppression system, the defection of p53 promotes the influence of genetic instability by G-quadruplex and i-motif (AMPARO et al., 2020), and the G-quadruplex structure impairs the transactivation of the target genes of p53α isoform (Porubiaková et al., 2019). The regulation network of p53 and G-quadruplex suggests potential modulation in more fields and this paper summarized their roles in the regulation of ferroptosis in cancer (Table 1).

**TABLE 1:** The regulation and mechanism in ferroptosis related to G-quadruplex and p53.

Process	Regulator	Targets	Mechanism	Reference
G-quadruplex-heme interaction	labile heme	G-quadruplex in myc promoter region	Labile heme binds to the G-quadruplex in the myc promoter region and blocks the transcription	Canesin et al. (2020)
	G-quadruplex	heme	G-quadruplex could bind or release heme in different conditions	Mestre-Fos et al. (2020); Gray et al. (2019)
iron-sulfur cluster biosynthesis	p53	iron-sulfur cluster assembly enzyme (ISCU)	P53 regulates ISCU and affects the biosynthesis of Fe-S cluster	Funauchi et al. (2015)
	Fe-S cluster helicase FANCJ	G-quadruplex	FANCJ unwinds G-quadruplex through Fe-S domain	Wu et al. (2008); London et al. (2008); Odermatt et al. (2020)
telomere homeostasis	telemore	p53	Telomere inactivation could activate p53	Sahin et al. (2011)
	p53	telemore	P53–p21–DREAM–E2F/CHR pathway down-regulates telomere maintenance	Engeland (2018); Vodicka et al. (2021)
	ROS	G-quadruplex	ROS in ferroptosis destroies G-quadruplex and affect telomere homeostasis	Ko et al. (2018); Kordowitzki (2021)
lipid peroxidation	MDM2	p53, PPARα	MDM2 promotes ferroptosis by p53 degradation and PPARα-mediated lipid remodeling	Bykov et al. (2018); Venkatesh et al. (2020)
	G-quadruplex	MDM2	G-quadruplex suppresses MDM2 transcription	Lago et al. (2021)
Glycolysis	p53	GLUT1, GLUT4, GLUT12	P53 inhibits the transcription of GLUT family, and imposes ferroptosis	Schwartzenberg-Bar-Yoseph et al. (2004); Zawacka-Pankau et al. (2011); Yokoyama et al. (2014)
	G-quadruplex	AMPK/SnRK, NrF2-related, and hypoxia-responsive transcription factors	G-quadruplex regulates transcription of target genes	Belmonte-Reche et al. (2022); Andorf et al. (2014)

## G-quadruplex interacts with ferric iron or heme

A mononuclear Fe (III) complex stabilizes G-quadruplex through π-π stacking and inhibits DNA amplification (Ebrahimi et al., 2015). As an essential regulatory factor, heme is originally considered as ferric ions storage and release pool (Daher et al., 2017; Fiorito et al., 2020), and in recent decades more studies display the heme function in cancer area (Gamage et al., 2021). Tumor cells employ heme to promote mitochondrial oxidative phosphorylation (OXPHOS) through the electron transport chain (ETC) (Hooda et al., 2013; Sugiyama et al., 2014; Alam et al., 2016; Fukuda et al., 2017; Ghosh et al., 2020). Heme binds to almost all parallel G-quadruplex structures to form a stable heme-G-quadruplex complex (Nakajima et al., 2022). Labile heme binds to the G-quadruplex in the myc promoter region and blocks the transcription, as well as the expression of myc downstream genes (Canesin et al., 2020). The G-quadruplexes formed in human ribosomes regulate heme bioavailability through binding to heme or releasing heme through a competitive ligand, PhenDC3 (Mestre-Fos et al., 2020), and further regulating genes related to ferroptosis (Gray et al., 2019). Moreover, G-quadruplex displays the function to be the labile heme pool *in vivo* (Kawai et al., 2022). Through dye-loaded hemin/G-quadruplex modification, the UiO-66 metal-organic framework nanoparticles can be used to detect microRNAs or genes including p53 and BRCA1 (Zhang et al., 2021). An extension of this study is the detection of more genes or RNAs based on sequence specificity. In brief, the G-quadruplex exhibits regulatory function through direct interaction with ferric ion or heme.

## G-quadruplex and p53 in ferroptosis

### G-quadruplex and iron-sulfur cluster biosynthesis in p53-regulated ferroptosis

The p53 participates in iron metabolism by regulating the transcriptional process of iron-sulfur cluster assembly enzyme (ISCU), and ISCU is critical for the biogenesis of iron-sulfur (Fe-S) cluster (Funauchi et al., 2015). The structure of the Fe-S cluster is first determined in the last century (Iwata et al., 1996) and it plays important role in cancer. In lung cancer, the overexpression of NFS1, one kind of iron-sulfur cluster biosynthetic enzyme, will sustain the iron-sulfur cluster expression, and inhibition of NFS1 leads to iron starvation and result in ferroptosis (Alvarez et al., 2017). In addition, sulfur transfer pathways also participate in the occurrence of ferroptosis (Yu et al., 2017). Fe-S cluster is involved in intracellular reduction/oxidation (REDOX) processes, FANCJ is one of the Fe-S cluster helicases, and the conserved Fe-S domain containing four cysteine residues is important for the cluster regulation (Bharti et al., 2013). Mutations in this FANCJ Fe-S domain would influence the cancer susceptibility (Paulo et al., 2018).

FANCJ is likely to be the only Fe-S cluster helicase to open the G-quadruplex structures to this day (Bharti et al., 2013). Human cells with FANCJ defection exhibit increased sensitivity to the G-quadruplex specific ligand (Wu et al., 2008), and FANCJ mutated cells derived from patients enrich in genome regions along with G-quadruplex structures (London et al., 2008). A series of mutation designs demonstrate that FANCJ unwinds G-quadruplex in the genome through the Fe-S domain, correlating with the ability of ferric iron incorporation and metabolism. Therefore, based on ferric iron regulation and ferroptosis, drugs targeting G-quadruplexes should be reconsidered in clinical use in cancer patients with FANCJ deficiency (Odermatt et al., 2020).

In a word, the Fe-S cluster helicase FANCJ utilizes the Fe-S domain to regulate G-quadruplex and likely further influence p53-related iron metabolism and ferroptosis, as well as affect genomic instability by unwinding G-quadruplexes in cancer.

### The p53-regulated telomere and the G-quadruplex function in ferroptosis

The regulatory relationship between telomere and p53 is complex. For example, telomere inactivation can activate p53, which leads to DNA damage and DNA repair at the end of chromosomes (Sahin et al., 2011), meanwhile, the p53–p21–DREAM–E2F/CHR pathway, in turn, down-regulates telomere maintenance and influence telomere homeostasis in cancer (Engeland, 2018; Vodicka et al., 2021).

As a signature of ferroptosis, ROS plays a crucial role in cancer. ROS could generate 8-oxoguanine through *in situ* oxidation of guanine in telomere, and this oxidative telomeric DNA damage, as well as the increased TERT expression, appears to be one of the most important causes of telomere shortening, resulting in increased mortality and cancer recurrence (Ko et al., 2018; Kordowitzki, 2021). RSL3-mediated oxidative stress in ferroptosis drives a series of histone modifications, and H3K79me3/H2A.Z could regulate the telomeric regions (Logie et al., 2021). Telomerase reverse transcriptase (TERT) is involved in ferroptosis-related differential expression genes, indicating potential regulation between telomere and ferroptosis (Liu H.-J. et al., 2020).

Telomeres are found to form G-quadruplex structures to inhibit DNA repair and sustain genome integrity. The ROS in ferroptosis will cause the replacement of guanine by 8-oxo-guanine and destroy the G-quadruplex structure, influencing genome instability and telomere homeostasis in cancer, which is also regulated by p53 pathways.

### G-quadruplex and lipid peroxidation in the p53-regulated ferroptosis

Accumulated iron triggers ferroptosis by producing excessive ROS and inducing lipid peroxidation (Liu et al., 2022; Ou et al., 2022; Qiao et al., 2022). As a critical component of the cellular antioxidant defense system, glutathione (GSH) prevents the accumulation of ROS and constitutes the major cellular defense mechanism against ferroptosis. GSH is synthesized from L-cysteine, L-glutamate, and glycine; therefore, the cellular availability of these amino acids could directly affect the concentration of GSH. Several studies have developed a strategy based on G-quadruplex formation for the detection of glutathione and cysteine in the biological sample (Leung et al., 2013; Zhao et al., 2013). These methods were developed to investigate G-quadruplex prevalence in human cells and to study their biological functions, presenting the next key challenges that need to be addressed to fully unravel their biology and therapeutic potential. MDM2 can target and degrade p53, while oncogene activation could prevent MDM2 from binding to p53 and stimulate the p53 acetylation (Bykov et al., 2018). MDM2 also can promote ferroptosis by PPARα-mediated lipid remodeling (Venkatesh et al., 2020). G-quadruplex structures are present in the MDM2 promoter and G-quadruplex ligands inhibit MDM2 expression and p53 degradation in the liposarcoma (Lago et al., 2021). Based on these published data, we have reasons to believe that G-quadruplex could be exploited to detect and modulate lipid peroxidation, probably reconstituting the p53-regulated ferroptosis signal.

### G-quadruplex involved in p53-regulated glycolysis and ferroptosis

Under most circumstances, p53 inhibits glucose uptake via direct attenuating glucose transporters glucose transporter 1 (GLUT1), GLUT4, and GLUT12 gene transcription and then drives glycolysis inhibition (Schwartzenberg-Bar-Yoseph et al., 2004; Zawacka-Pankau et al., 2011; Yokoyama et al., 2014). Glucose-metabolism imbalance would activate the LKB1/AMPK regulatory axis to cause the phosphorylation of acetyl-CoA carboxylase (ACC) to inhibit its activity and impose a regulatory effect on tumor cell ferroptosis (Lee et al., 2020; Li et al., 2020). Glycosyl conjugation to drugs is a strategy being used to take advantage of GLUT overexpression in cancer cells in comparison with non-cancerous cells. Efres *et.al* have synthesized thiosugar naphthalene diimide conjugates as G-quadruplex ligand and proved their antiproliferative activity in colon cancer cells (Belmonte-Reche et al., 2022). Furthermore, G-quadruplex motifs are found in numerous genes encoding members of the AMPK/SnRK, NrF2-realated, and hypoxia-responsive transcription factors (Andorf et al., 2014). Collectively, G-quadruplex may aid in energy status gene responses and provide a mechanistic basis for linking Glycolysis signals to ferroptosis.

## Conclusion and perspectives

Ferroptosis is a new regulated cell death form and the mechanisms in cancer are still under exploration. As important regulatory elements, both G-quadruplex and p53 are involved in various ferroptosis-related processes, and the potential diversified interplay provides more understanding of ferric ion/heme, Fe-S cluster biosynthesis, telomere homeostasis, lipid peroxidation, and glucose metabolism.

The classic regulatory mode of p53 contains stabilization, antirepression, and promoter-specific activation (Kruse and Gu, 2009), and recent research has highlighted the importance of the posttranslational modifications (Liu et al., 2019; Zhang L. et al., 2022). The p53 participates in regulating iron-sulfur cluster assembly enzyme activity, interacts with telomere, is involved in lipid peroxidation, and regulates glycolysis. The complicated models are tightly involved in p53-mediated ferroptosis. Targeting p53 pathways is a promising strategy for anticancer therapy, and various inhibitors are being developed, including ZNF498 (Zhang X. et al., 2022), and Eupaformosanin (Wei et al., 2022).

G-quadruplex acts as a vital regulator for gene activity based on its biological function thus attracting great enthusiasm from researchers in the field of drug discovery. G-quadruplex binding ligands, mostly small molecules, change the stabilization of this kind of secondary structure and further affect the gene activity, telomeric function, and genome instability in multiple cancers. Many small molecules have been discovered and several molecules have progressed to clinical trials, such as Quarfloxin/CX-3534 (Phase I/II) targeting different cancers (NCT00780663, NCT00955786) (Papadopoulos et al., 2007; Drygin et al., 2009), and CX-5461 (Phase I) targeting BRCA1/2 deficient cancer (NCT02719977) (Xu et al., 2017; Khot et al., 2019; Hilton et al., 2022).

**FIGURE 2:**
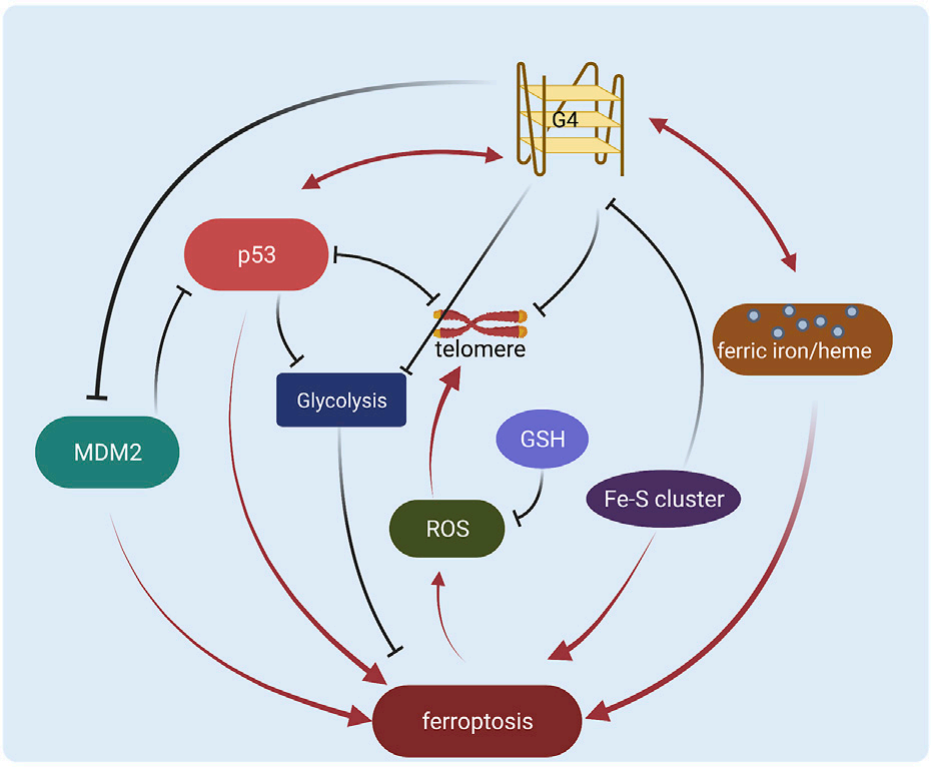
Schematic diagram of the regulation network of G-quadruplex and p53 in ferroptosis (Created with BioRender.com).

However, the G-quadruplex regulation in cancer still remains unsolved problems. Many G-quadruplex ligands don’t exhibit selectivity between different G-quadruplexes (Chen J. et al., 2021; Galati et al., 2021), generating potential side effects or low efficiency. The binding affinity of some ligands still needs to be improved (Kosiol et al., 2021) to benefit clinical usage. The regulation of G-quadruplex and ligand function in cancer is yet not clear. Therefore it is necessary to explore new modulations, such as the direct/indirect interactions with p53 in ferroptosis regulation. Here we show that p53 might be an important regulator or target of the G-quadruplex, especially in ferroptosis of cancer research (Figure 2). It is still a challenge to figure out how p53 and G-quadruplex interplay in more stages such as posttranslational modifications or DNA binding, and if the crosstalk functions in other processes like cell cycle/apoptosis. More potential interactions need to be characterized with high resolution, thus methodologies including computational simulation and experimental tools are also required for robust molecular exploration. In addition, it will be interesting and crucial to study the direct interactions between p53 and G-quadruplex in the regulation of ferroptosis in cancer to achieve a clearer mechanism. Besides, diverse studies regarding p53 and G-quadruplex in ferroptosis are operated *in vitro*, and more *in vivo* validations are essential for future therapeutic investigations. Furthermore, more specific targeting strategies are required to evolve based on the potential interplay between p53 and G-quadruplex.

In conclusion, the G-quadruplex and p53 regulation network might be a potential target for cancer research in the future and the mechanisms will be better understood as the research attention increases, hopefully benefiting the clinical cancer treatment.

## References

[B1] Adámik M. Kejnovská I. Bažantová P. Petr M. Renčiuk D. Vorlíčková M. (2016). p53 binds human telomeric G-quadruplex *in vitro* . Biochimie 128-129, 83–91. 10.1016/j.biochi.2016.07.004 27422117

[B2] Alam M. M. Lal S. FitzGerald K. E. Zhang L. (2016). A holistic view of cancer bioenergetics: Mitochondrial function and respiration play fundamental roles in the development and progression of diverse tumors. Clin. Transl. Med. 5 (1), 3. 10.1186/s40169-016-0082-9 26812134 PMC4728164

[B3] Alvarez S. W. Sviderskiy V. O. Terzi E. M. Papagiannakopoulos T. Moreira A. L. Adams S. (2017). NFS1 undergoes positive selection in lung tumours and protects cells from ferroptosis. Nature 551 (7682), 639–643. 10.1038/nature24637 29168506 PMC5808442

[B4] Amparo C. Clark J. Bedell V. Murata-Collins J. L. Martella M. Pichiorri F. (2020). Duplex DNA from sites of helicase-polymerase Uncoupling Links non-B DNA structure formation to replicative stress. Cancer Genomics Proteomics 17 (2), 101–115. 10.21873/cgp.20171 32108033 PMC7078840

[B5] Andorf C. M. Kopylov M. Dobbs D. Koch K. E. Stroupe M. E. Lawrence C. J. (2014). G-quadruplex (G4) motifs in the maize (Zea mays L.) genome are enriched at specific Locations in Thousands of genes Coupled to energy status, hypoxia, low sugar, and Nutrient Deprivation. J. Genet. Genomics 41 (12), 627–647. 10.1016/j.jgg.2014.10.004 25527104

[B6] Beniaminov A. D. Novikov R. A. Mamaeva O. K. Mitkevich V. A. Smirnov I. P. Livshits M. A. (2016). Light-induced oxidation of the telomeric G4 DNA in complex with Zn(II) tetracarboxymethyl porphyrin. Nucleic Acids Res. 44 (21), 10031–10041. 10.1093/nar/gkw947 27915287 PMC5137456

[B7] Beauvarlet J. Bensadoun P. Darbo E. Labrunie G. Rousseau B. Richard E. (2019). Modulation of the ATM/autophagy pathway by a G-quadruplex ligand tips the balance between senescence and apoptosis in cancer cells. Nucleic Acids Res. 47 (6), 2739–2756. 10.1093/nar/gkz095 30759257 PMC6451122

[B8] Belmonte-Reche E. Benassi A. Peñalver P. Cucchiarini A. Guédin A. Mergny J. L. (2022). Thiosugar naphthalene diimide conjugates: G-Quadruplex ligands with antiparasitic and anticancer activity. Eur. J. Med. Chem. 232, 114183. 10.1016/j.ejmech.2022.114183 35168151

[B9] Bharti S. K. Sommers J. A. George F. Kuper J. Hamon F. Shin-ya K. (2013). Specialization among iron-sulfur cluster helicases to resolve G-quadruplex DNA structures that threaten genomic stability. J. Biol. Chem. 288 (39), 28217–28229. 10.1074/jbc.M113.496463 23935105 PMC3784731

[B10] Bykov V. J. N. Eriksson S. E. Bianchi J. Wiman K. G. (2018). Targeting mutant p53 for efficient cancer therapy. Nat. Rev. Cancer 18 (2), 89–102. 10.1038/nrc.2017.109 29242642

[B11] Bywater M. J. Poortinga G. Sanij E. Hein N. Peck A. Cullinane C. (2012). Inhibition of RNA polymerase I as a therapeutic strategy to promote cancer-specific activation of p53. Cancer Cell 22 (1), 51–65. 10.1016/j.ccr.2012.05.019 22789538 PMC3749732

[B12] Canesin G. Di Ruscio A. Li M. Ummarino S. Hedblom A. Choudhury R. (2020). Scavenging of labile heme by Hemopexin is a key Checkpoint in cancer Growth and Metastases. Cell Rep. 32 (12), 108181. 10.1016/j.celrep.2020.108181 32966797 PMC7551404

[B13] Chen D. Chu B. Yang X. Liu Z. Jin Y. Kon N. (2021a). iPLA2β-mediated lipid detoxification controls p53-driven ferroptosis independent of GPX4. Nat. Commun. 12 (1), 3644. 10.1038/s41467-021-23902-6 34131139 PMC8206155

[B14] Chen J. Jin X. Mei Y. Shen Z. Zhu J. Shi H. (2021b). The different biological effects of TMPyP4 and cisplatin in the inflammatory microenvironment of osteosarcoma are attributed to G-quadruplex. Cell Prolif. 54 (9), e13101. 10.1111/cpr.13101 34296479 PMC8450119

[B15] Chen X. Kang R. Kroemer G. Tang D. (2021c). Broadening horizons: The role of ferroptosis in cancer. Nat. Rev. Clin. Oncol. 18 (5), 280–296. 10.1038/s41571-020-00462-0 33514910

[B16] Chu B. Kon N. Chen D. Li T. Liu T. Jiang L. (2019). ALOX12 is required for p53-mediated tumour suppression through a distinct ferroptosis pathway. Nat. Cell Biol. 21 (5), 579–591. 10.1038/s41556-019-0305-6 30962574 PMC6624840

[B17] Daher R. Manceau H. Karim Z. (2017). Iron metabolism and the role of the iron-regulating hormone hepcidin in health and disease. Presse Med. 46 (12 Pt 2), e272–e278. 10.1016/j.lpm.2017.10.006 29129410

[B18] Dixon S. J. Lemberg K. M. Lamprecht M. R. Skouta R. Zaitsev E. M. Gleason C. E. (2012). Ferroptosis: An iron-dependent form of nonapoptotic cell death. Cell 149 (5), 1060–1072. 10.1016/j.cell.2012.03.042 22632970 PMC3367386

[B19] Drygin D. Siddiqui-Jain A. O'Brien S. Schwaebe M. Lin A. Bliesath J. (2009). Anticancer activity of CX-3543: A direct inhibitor of rRNA biogenesis. Cancer Res. 69 (19), 7653–7661. 10.1158/0008-5472.Can-09-1304 19738048

[B20] Ebrahimi M. Khayamian T. Hadadzadeh H. Sayed Tabatabaei B. E. Jannesari Z. Khaksar G. (2015). Spectroscopic, biological, and molecular modeling studies on the interactions of [Fe(III)-meloxicam] with G-quadruplex DNA and investigation of its release from bovine serum albumin (BSA) nanoparticles. J. Biomol. Struct. Dyn. 33 (11), 2316–2329. 10.1080/07391102.2014.1003195 25563680

[B21] Engeland K. (2018). Cell cycle arrest through indirect transcriptional repression by p53: I have a DREAM. Cell Death Differ. 25 (1), 114–132. 10.1038/cdd.2017.172 29125603 PMC5729532

[B22] Fiorito V. Chiabrando D. Petrillo S. Bertino F. Tolosano E. (2020). The Multifaceted role of heme in cancer. Front. Oncol. 9, 1540. 10.3389/fonc.2019.01540 32010627 PMC6974621

[B23] Fukuda Y. Wang Y. Lian S. Lynch J. Nagai S. Fanshawe B. (2017). Upregulated heme biosynthesis, an exploitable vulnerability in MYCN-driven leukemogenesis. JCI Insight 2 (15), 92409. 10.1172/jci.insight.92409 28768907 PMC5543914

[B24] Funauchi Y. Tanikawa C. Yi Lo P. H. Mori J. Daigo Y. Takano A. (2015). Regulation of iron homeostasis by the p53-ISCU pathway. Sci. Rep. 5, 16497. 10.1038/srep16497 26560363 PMC4642350

[B25] Galati E. Bosio M. C. Novarina D. Chiara M. Bernini G. M. Mozzarelli A. M. (2021). VID22 counteracts G-quadruplex-induced genome instability. Nucleic Acids Res. 49 (22), 12785–12804. 10.1093/nar/gkab1156 34871443 PMC8682794

[B26] Gamage S. M. K. Lee K. T. W. Dissabandara D. L. O. Lam A. K. Gopalan V. (2021). Dual role of heme iron in cancer; promotor of carcinogenesis and an inducer of tumour suppression. Exp. Mol. Pathol. 120, 104642. 10.1016/j.yexmp.2021.104642 33905708

[B27] Ghosh P. Vidal C. Dey S. Zhang L. (2020). Mitochondria targeting as an effective strategy for cancer therapy. Int. J. Mol. Sci. 21 (9), 3363. 10.3390/ijms21093363 PMC7247703 32397535

[B28] Gray L. T. Puig Lombardi E. Verga D. Nicolas A. Teulade-Fichou M. P. Londoño-Vallejo A. (2019). G-Quadruplexes Sequester free heme in Living cells. Cell Chem. Biol. 26 (12), 1681–1691. e1685. 10.1016/j.chembiol.2019.10.003 31668518

[B29] Hänsel-Hertsch R. Beraldi D. Lensing S. V. Marsico G. Zyner K. Parry A. (2016). G-quadruplex structures mark human regulatory chromatin. Nat. Genet. 48 (10), 1267–1272. 10.1038/ng.3662 27618450

[B30] Hilton J. Gelmon K. Bedard P. L. Tu D. Xu H. Tinker A. V. (2022). Results of the phase I CCTG IND.231 trial of CX-5461 in patients with advanced solid tumors enriched for DNA-repair deficiencies. Nat. Commun. 13 (1), 3607. 10.1038/s41467-022-31199-2 35750695 PMC9232501

[B31] Hooda J. Cadinu D. Alam M. M. Shah A. Cao T. M. Sullivan L. A. (2013). Enhanced heme function and mitochondrial respiration promote the progression of lung cancer cells. PLoS One 8 (5), e63402. 10.1371/journal.pone.0063402 23704904 PMC3660535

[B32] Iwata S. Saynovits M. Link T. A. Michel H. (1996). Structure of a water soluble fragment of the 'Rieske' iron-sulfur protein of the bovine heart mitochondrial cytochrome bc1 complex determined by MAD phasing at 1.5 A resolution. Structure 4 (5), 567–579. 10.1016/s0969-2126(96)00062-7 8736555

[B33] Jiang L. Kon N. Li T. Wang S. J. Su T. Hibshoosh H. (2015). Ferroptosis as a p53-mediated activity during tumour suppression. Nature 520 (7545), 57–62. 10.1038/nature14344 25799988 PMC4455927

[B34] Kang R. Kroemer G. Tang D. (2019). The tumor suppressor protein p53 and the ferroptosis network. Free Radic. Biol. Med. 133, 162–168. 10.1016/j.freeradbiomed.2018.05.074 29800655 PMC6251771

[B35] Kawai K. Hirayama T. Imai H. Murakami T. Inden M. Hozumi I. (2022). Molecular Imaging of labile heme in Living cells using a small molecule fluorescent probe. J. Am. Chem. Soc. 144 (9), 3793–3803. 10.1021/jacs.1c08485 35133144

[B36] Khot A. Brajanovski N. Cameron D. P. Hein N. Maclachlan K. H. Sanij E. (2019). First-in-Human RNA polymerase I transcription inhibitor CX-5461 in patients with advanced Hematologic cancers: Results of a phase I Dose-Escalation study. Cancer Discov. 9 (8), 1036–1049. 10.1158/2159-8290.Cd-18-1455 31092402

[B37] Ko E. Seo H.-W. Jung G. (2018). Telomere length and reactive oxygen species levels are positively associated with a high risk of mortality and recurrence in hepatocellular carcinoma. Hepatology 67 (4), 1378–1391. 10.1002/hep.29604 29059467

[B38] Kordowitzki P. (2021). Oxidative stress induces telomere dysfunction and shortening in human Oocytes of advanced Age Donors. Cells 10 (8), 1866. 10.3390/cells10081866 34440635 PMC8391391

[B39] Kosiol N. Juranek S. Brossart P. Heine A. Paeschke K. (2021). G-Quadruplexes: A promising target for cancer therapy. Mol. Cancer 20 (1), 40. 10.1186/s12943-021-01328-4 33632214 PMC7905668

[B40] Kruse J. P. Gu W. (2009). Modes of p53 regulation. Cell 137 (4), 609–622. 10.1016/j.cell.2009.04.050 19450511 PMC3737742

[B41] Lago S. Nadai M. Ruggiero E. Tassinari M. Marušič M. Tosoni B. (2021). The MDM2 inducible promoter folds into four-tetrad antiparallel G-quadruplexes targetable to fight malignant liposarcoma. Nucleic Acids Res. 49 (2), 847–863. 10.1093/nar/gkaa1273 33410915 PMC7826256

[B42] Lee H. Zhuang L. Gan B. (2020). Energy stress inhibits ferroptosis via AMPK. Mol. Cell. Oncol. 7 (4), 1761242. 10.1080/23723556.2020.1761242 32944623 PMC7469505

[B43] Leung K. H. He H. Z. Ma V. P. Chan D. S. Leung C. H. Ma D. L. (2013). A luminescent G-quadruplex switch-on probe for the highly selective and tunable detection of cysteine and glutathione. Chem. Commun. 49 (8), 771–773. 10.1039/c2cc37710a 23192322

[B44] Li C. Dong X. Du W. Shi X. Chen K. Zhang W. (2020). LKB1-AMPK axis negatively regulates ferroptosis by inhibiting fatty acid synthesis. Signal Transduct. Target. Ther. 5 (1), 187. 10.1038/s41392-020-00297-2 32883948 PMC7471309

[B45] Liu H.-J. Hu H.-M. Li G.-Z. Zhang Y. Wu F. Liu X. (2020a). Ferroptosis-related gene signature Predicts Glioma cell death and Glioma patient progression. Front. Cell Dev. Biol. 8, 538. 10.3389/fcell.2020.00538 32733879 PMC7363771

[B46] Liu J. Zhang C. Wang J. Hu W. Feng Z. (2020b). The regulation of ferroptosis by tumor suppressor p53 and its pathway. Int. J. Mol. Sci. 21 (21), E8387. 10.3390/ijms21218387 33182266 PMC7664917

[B47] Liu M. Kong X. Y. Yao Y. Wang X. A. Yang W. Wu H. (2022). The critical role and molecular mechanisms of ferroptosis in antioxidant systems: A narrative review. Ann. Transl. Med. 10 (6), 368. 10.21037/atm-21-6942 35434035 PMC9011221

[B48] Liu Y. Gu W. (2022). p53 in ferroptosis regulation: the new weapon for the old guardian. Cell Death Differ. 29 (5), 895–910. 10.1038/s41418-022-00943-y 35087226 PMC9091200

[B49] Liu Y. Gu W. (2021). The complexity of p53-mediated metabolic regulation in tumor suppression. Semin. Cancer Biol. 10.1016/j.semcancer.2021.03.010 PMC8473587 33785447

[B50] Liu Y. Tavana O. Gu W. (2019). p53 modifications: exquisite decorations of the powerful guardian. J. Mol. Cell Biol. 11 (7), 564–577. 10.1093/jmcb/mjz060 31282934 PMC6736412

[B51] Logie E. Van Puyvelde B. Cuypers B. Schepers A. Berghmans H. Verdonck J. (2021). Ferroptosis induction in multiple Myeloma cells triggers DNA Methylation and histone modification changes associated with cellular senescence. Int. J. Mol. Sci. 22 (22), 12234. 10.3390/ijms222212234 34830117 PMC8618106

[B52] London T. B. Barber L. J. Mosedale G. Kelly G. P. Balasubramanian S. Hickson I. D. (2008). FANCJ is a structure-specific DNA helicase associated with the maintenance of genomic G/C tracts. J. Biol. Chem. 283 (52), 36132–36139. 10.1074/jbc.M808152200 18978354 PMC2662291

[B53] Marcel V. Tran P. L. T. Sagne C. Martel-Planche G. Vaslin L. Teulade-Fichou M.-P. (2010). G-Quadruplex structures in TP53 intron 3: Role in alternative splicing and in production of p53 mRNA isoforms. Carcinogenesis 32 (3), 271–278. 10.1093/carcin/bgq253 21112961

[B54] Mestre-Fos S. Ito C. Moore C. M. Reddi A. R. Williams L. D. (2020). Human ribosomal G-quadruplexes regulate heme bioavailability. J. Biol. Chem. 295 (44), 14855–14865. 10.1074/jbc.RA120.014332 32817343 PMC7606673

[B55] Nakajima Y. Momotake A. Suzuki A. Neya S. Yamamoto Y. (2022). Nature of a H2O molecule Confined in the Hydrophobic Interface between the heme and G-quartet Planes in a heme–DNA complex. Biochemistry 61 (7), 523–534. 10.1021/acs.biochem.1c00751 35230084

[B56] Nasiri H. R. Bell N. M. McLuckie K. I. Husby J. Abell C. Neidle S. (2014). Targeting a c-MYC G-quadruplex DNA with a fragment library. Chem. Commun. 50 (14), 1704–1707. 10.1039/c3cc48390h 24394582

[B57] Newman M. Sfaxi R. Saha A. Monchaud D. Teulade-Fichou M. P. Vagner S. (2017). The G-quadruplex-specific RNA helicase DHX36 regulates p53 pre-mRNA 3'-end processing following UV-induced DNA damage. J. Mol. Biol. 429 (21), 3121–3131. 10.1016/j.jmb.2016.11.033 27940037

[B58] Odermatt D. C. Lee W. T. C. Wild S. Jozwiakowski S. K. Rothenberg E. Gari K. (2020). Cancer-associated mutations in the iron-sulfur domain of FANCJ affect G-quadruplex metabolism. PLoS Genet. 16 (6), e1008740. 10.1371/journal.pgen.1008740 32542039 PMC7316351

[B59] Ou M. Jiang Y. Ji Y. Zhou Q. Du Z. Zhu H. (2022). Role and mechanism of ferroptosis in neurological diseases. Mol. Metab. 61, 101502. 10.1016/j.molmet.2022.101502 35447365 PMC9170779

[B60] Ou Y. Wang S. J. Li D. Chu B. Gu W. (2016). Activation of SAT1 engages polyamine metabolism with p53-mediated ferroptotic responses. Proc. Natl. Acad. Sci. U. S. A. 113 (44), E6806–E6812. 10.1073/pnas.1607152113 27698118 PMC5098629

[B61] Papadopoulos K. Mita A. Ricart A. Hufnagel D. Northfelt D. Von Hoff D. (2007). Pharmacokinetic findings from the phase I study of Quarfloxin (CX-3543): A protein-rDNA quadruplex inhibitor, in patients with advanced solid tumors. Mol. Cancer Ther. 6 (11_Suppl. ment), B93.

[B62] Paulo P. Maia S. Pinto C. Pinto P. Monteiro A. Peixoto A. (2018). Targeted next generation sequencing identifies functionally deleterious germline mutations in novel genes in early-onset/familial prostate cancer. PLoS Genet. 14 (4), e1007355. 10.1371/journal.pgen.1007355 29659569 PMC5919682

[B63] Petr M. Helma R. Polášková A. Krejčí A. Dvořáková Z. Kejnovská I. (2016). Wild-type p53 binds to MYC promoter G-quadruplex. Biosci. Rep. 36 (5), e00397. 10.1042/BSR20160232 27634752 PMC5064454

[B64] Porubiaková O. Bohálová N. Inga A. Vadovičová N. Coufal J. Fojta M. (2019). The influence of quadruplex structure in Proximity to P53 target sequences on the transactivation potential of P53 Alpha isoforms. Int. J. Mol. Sci. 21 (1), 127. 10.3390/ijms21010127 PMC6982142 31878115

[B65] Qiao G. Zhang W. Dong K. (2022). Regulation of ferroptosis by noncoding RNAs: A novel promise treatment in esophageal squamous cell carcinoma. Mol. Cell. Biochem. 10.1007/s11010-022-04441-0 35449482

[B66] Rhodes D. Lipps H. J. (2015). G-quadruplexes and their regulatory roles in biology. Nucleic Acids Res. 43 (18), 8627–8637. 10.1093/nar/gkv862 26350216 PMC4605312

[B67] Sahin E. Colla S. Liesa M. Moslehi J. Müller F. L. Guo M. (2011). Telomere dysfunction induces metabolic and mitochondrial compromise. Nature 470 (7334), 359–365. 10.1038/nature09787 21307849 PMC3741661

[B68] Schwartzenberg-Bar-Yoseph F. Armoni M. Karnieli E. (2004). The tumor suppressor p53 down-regulates glucose transporters GLUT1 and GLUT4 gene expression. Cancer Res. 64 (7), 2627–2633. 10.1158/0008-5472.can-03-0846 15059920

[B69] Shen J. Varshney D. Simeone A. Zhang X. Adhikari S. Tannahill D. (2021). Promoter G-quadruplex folding precedes transcription and is controlled by chromatin. Genome Biol. 22 (1), 143. 10.1186/s13059-021-02346-7 33962653 PMC8103603

[B70] Siddiqui-Jain A. Grand C. L. Bearss D. J. Hurley L. H. (2002). Direct evidence for a G-quadruplex in a promoter region and its targeting with a small molecule to repress c-MYC transcription. Proc. Natl. Acad. Sci. U. S. A. 99 (18), 11593–11598. 10.1073/pnas.182256799 12195017 PMC129314

[B71] Spiegel J. Adhikari S. Balasubramanian S. (2020). The structure and function of DNA G-quadruplexes. Trends Chem. 2 (2), 123–136. 10.1016/j.trechm.2019.07.002 32923997 PMC7472594

[B72] Sugiyama Y. Hagiya Y. Nakajima M. Ishizuka M. Tanaka T. Ogura S. (2014). The heme precursor 5-aminolevulinic acid disrupts the Warburg effect in tumor cells and induces caspase-dependent apoptosis. Oncol. Rep. 31 (3), 1282–1286. 10.3892/or.2013.2945 24366173

[B73] Tarangelo A. Magtanong L. Bieging-Rolett K. T. Li Y. Ye J. Attardi L. D. (2018). p53 suppresses metabolic stress-induced ferroptosis in cancer cells. Cell Rep. 22 (3), 569–575. 10.1016/j.celrep.2017.12.077 29346757 PMC5791910

[B74] Tauchi T. Shin-Ya K. Sashida G. Sumi M. Nakajima A. Shimamoto T. (2003). Activity of a novel G-quadruplex-interactive telomerase inhibitor, telomestatin (SOT-095), against human leukemia cells: Involvement of ATM-dependent DNA damage response pathways. Oncogene 22 (34), 5338–5347. 10.1038/sj.onc.1206833 12917635

[B75] Varshney D. Spiegel J. Zyner K. Tannahill D. Balasubramanian S. (2020). The regulation and functions of DNA and RNA G-quadruplexes. Nat. Rev. Mol. Cell Biol. 21 (8), 459–474. 10.1038/s41580-020-0236-x 32313204 PMC7115845

[B76] Venkatesh D. O'Brien N. A. Zandkarimi F. Tong D. R. Stokes M. E. Dunn D. E. (2020). MDM2 and MDMX promote ferroptosis by PPARα-mediated lipid remodeling. Genes Dev. 34 (7-8), 526–543. 10.1101/gad.334219.119 32079652 PMC7111265

[B77] Vodicka P. Andera L. Opattova A. Vodickova L. (2021). The interactions of DNA repair, telomere homeostasis, and p53 mutational status in solid cancers: Risk, Prognosis, and Prediction. Cancers (Basel) 13 (3), 479. 10.3390/cancers13030479 33513745 PMC7865496

[B78] Wang M. Mao C. Ouyang L. Liu Y. Lai W. Liu N. (2019). Long noncoding RNA LINC00336 inhibits ferroptosis in lung cancer by functioning as a competing endogenous RNA. Cell Death Differ. 26 (11), 2329–2343. 10.1038/s41418-019-0304-y 30787392 PMC6889193

[B79] Wei Y. Zhu Z. Hu H. Guan J. Yang B. Zhao H. (2022). Eupaformosanin induces apoptosis and ferroptosis through ubiquitination of mutant p53 in triple-negative breast cancer. Eur. J. Pharmacol. 924, 174970. 10.1016/j.ejphar.2022.174970 35469839

[B80] Wu Y. Shin-ya K. Brosh R. M. Jr. (2008). FANCJ helicase defective in Fanconia anemia and breast cancer unwinds G-quadruplex DNA to defend genomic stability. Mol. Cell. Biol. 28 (12), 4116–4128. 10.1128/mcb.02210-07 18426915 PMC2423121

[B81] Xie Y. Zhu S. Song X. Sun X. Fan Y. Liu J. (2017). The tumor suppressor p53 Limits ferroptosis by blocking DPP4 activity. Cell Rep. 20 (7), 1692–1704. 10.1016/j.celrep.2017.07.055 28813679

[B82] Xu H. Di Antonio M. McKinney S. Mathew V. Ho B. O'Neil N. J. (2017). CX-5461 is a DNA G-quadruplex stabilizer with selective lethality in BRCA1/2 deficient tumours. Nat. Commun. 8, 14432. 10.1038/ncomms14432 28211448 PMC5321743

[B83] Yokoyama M. Okada S. Nakagomi A. Moriya J. Shimizu I. Nojima A. (2014). Inhibition of endothelial p53 improves metabolic abnormalities related to dietary obesity. Cell Rep. 7 (5), 1691–1703. 10.1016/j.celrep.2014.04.046 24857662

[B84] Yu H. Guo P. Xie X. Wang Y. Chen G. (2017). Ferroptosis, a new form of cell death, and its relationships with tumourous diseases. J. Cell. Mol. Med. 21 (4), 648–657. 10.1111/jcmm.13008 27860262 PMC5345622

[B85] Yu L. Wu M. Zhu G. Xu Y. (2021). Emerging roles of the tumor suppressor p53 in metabolism. Front. Cell Dev. Biol. 9, 762742. 10.3389/fcell.2021.762742 35118064 PMC8806078

[B86] Yuan G. Zhang Q. Zhou J. Li H. (2011). Mass spectrometry of G-quadruplex DNA: Formation, recognition, property, conversion, and conformation. Mass Spectrom. Rev. 30 (6), 1121–1142. 10.1002/mas.20315 21520218

[B87] Zawacka-Pankau J. Grinkevich V. V. Hünten S. Nikulenkov F. Gluch A. Li H. (2011). Inhibition of glycolytic enzymes mediated by pharmacologically activated p53: Targeting Warburg effect to fight cancer. J. Biol. Chem. 286 (48), 41600–41615. 10.1074/jbc.M111.240812 21862591 PMC3308870

[B88] Zhang L. Hou N. Chen B. Kan C. Han F. Zhang J. (2022a). Post-translational modifications of p53 in ferroptosis: Novel Pharmacological targets for cancer therapy. Front. Pharmacol. 13, 908772. 10.3389/fphar.2022.908772 35685623 PMC9171069

[B89] Zhang L. Tan W. Zhou J. Xu M. Yuan G. (2017). Investigation of G-quadruplex formation in the FGFR2 promoter region and its transcriptional regulation by liensinine. Biochim. Biophys. Acta. Gen. Subj. 1861 (4), 884–891. 10.1016/j.bbagen.2017.01.028 28132898

[B90] Zhang P. Ouyang Y. Willner I. (2021). Multiplexed and amplified chemiluminescence resonance energy transfer (CRET) detection of genes and microRNAs using dye-loaded hemin/G-quadruplex-modified UiO-66 metal-organic framework nanoparticles. Chem. Sci. 12 (13), 4810–4818. 10.1039/d0sc06744j 34163734 PMC8179566

[B91] Zhang W. Gai C. Ding D. Wang F. Li W. (2018). Targeted p53 on small-molecules-induced ferroptosis in cancers. Front. Oncol. 8, 507. 10.3389/fonc.2018.00507 30450337 PMC6224449

[B92] Zhang X. Zheng Q. Yue X. Yuan Z. Ling J. Yuan Y. (2022b). ZNF498 promotes hepatocellular carcinogenesis by suppressing p53-mediated apoptosis and ferroptosis via the attenuation of p53 Ser46 phosphorylation. J. Exp. Clin. Cancer Res. 41 (1), 79. 10.1186/s13046-022-02288-3 35227287 PMC8883630

[B93] Zhao J. Chen C. Zhang L. Jiang J. Shen G. Yu R. (2013). A Hg(2+)-mediated label-free fluorescent sensing strategy based on G-quadruplex formation for selective detection of glutathione and cysteine. Analyst 138 (6), 1713–1718. 10.1039/c3an36657j 23377184

[B94] Zhu L. N. Zhao S. J. Wu B. Li X. Z. Kong D. M. (2012). A new cationic porphyrin derivative (TMPipEOPP) with large side arm substituents: A highly selective G-quadruplex optical probe. PLoS One 7 (5), e35586. 10.1371/journal.pone.0035586 22629300 PMC3358308

